# Effects of heavy-load resistance training during (neo-)adjuvant chemotherapy on muscle cellular outcomes in women with breast cancer

**DOI:** 10.1097/MD.0000000000024960

**Published:** 2021-03-12

**Authors:** Emelie Strandberg, Karianne Vassbakk-Svindland, Anna Henriksson, Birgitta Johansson, Olav Vikmoen, David Kudrén, Tim Schauer, Henrik Lindman, Fredrik Wärnberg, Sveinung Berntsen, Ingrid Demmelmaier, Karin Nordin, Truls Raastad

**Affiliations:** a Department of Public Health and Caring Sciences; b Department of Immunology, Genetics and Pathology, Uppsala University, Uppsala, Sweden; c Department of Physical Performance, Norwegian School of Sport Science, Oslo, Norway; d Centre for Physical Activity Research, Rigshospitalet, University of Copenhagen, Copenhagen, Denmark; e Department of Oncology, Uppsala University Hospital, Uppsala; f Department of Surgery, Institute of Clinical Sciences, Sahlgrenska Academy, University of Gothenburg, Gothenburg, Sweden; g Department of Sport Science and Physical Education, University of Agder, Kristiansand, Norway.

**Keywords:** cancer, exercise, muscle fiber cross-sectional area, oncology, strength training

## Abstract

**Introduction:**

(Neo-)adjuvant chemotherapy for breast cancer has a deleterious impact on muscle tissue resulting in reduced cardiorespiratory fitness, skeletal muscle mass and function. Physical exercise during treatment may counteract some of these negative effects. However, the effects of resistance training (RT) alone have never been explored. The present study aims to investigate if heavy-load RT during (neo-)adjuvant chemotherapy counteracts deleterious effects on skeletal muscle in women diagnosed with breast cancer. We hypothesize that (neo-)adjuvant treatment with chemotherapy will reduce muscle fiber size, impair mitochondrial function, and increase indicators of cellular stress and that RT during treatment will counteract these negative effects. We also hypothesize that RT during (neo-)adjuvant chemotherapy will increase muscle and blood levels of potential antitumor myokines and reduce treatment-related side effects on muscle strength and cardiorespiratory fitness.

**Methods:**

Fifty women recently diagnosed with breast cancer scheduled to start (neo-)adjuvant chemotherapy will be randomized to either randomized to either intervention group or to control group.

The intervention group will perform supervised heavy-load RT twice a week over the course of chemotherapy (approximately 16-weeks) whereas the control group will be encouraged to continue with their usual activities. Muscle biopsies from *m. vastus lateralis* will be collected before the first cycle of chemotherapy (T0), after chemotherapy (T1), and 6 months later (T2) for assessment of muscle cellular outcomes. The primary outcome for this study is muscle fiber size. Secondary outcomes are: regulators of muscle fiber size and function, indicators of cellular stress and mitochondrial function, myokines with potential antitumor effects, muscle strength, and cardiorespiratory fitness.

**Ethics and dissemination:**

Ethical approval has been obtained from the Regional Ethical Review Board in Uppsala, Sweden (Dnr:2016/230/2). Results will be disseminated through presentations at scientific meetings, publications in peer-reviewed journals, social media, and patient organizations.

**Trial registration number:**

NCT04586517.

## 1 Introduction

The increasing survival rates of women diagnosed with breast cancer requires an increased focus on measures to counteract persistent adverse effects of treatment. Neo-adjuvant treatment aims to decrease tumor burden before surgery and adjuvant treatment to inhibit proliferation in eventual remaining cancer cells after surgery. A common (neo-)adjuvant treatment for women with breast cancer is combined chemotherapy with anthracyclines and taxanes, given in 3 cycles of each chemotherapy type with a recovery period after each cycle.^[1]^ Anthracyclines have a cardiotoxic effect^[2,3]^ and previous observational studies have shown approximately 10% decrease in maximal oxygen uptake (VO_2max_) during chemotherapy.^[4]^ VO_2max_ is determined by both central- (e.g., cardiac function) and peripheral factors (e.g., skeletal muscle function) and reductions in VO_2max_ are common in patients with breast cancer without any signs of impaired cardiac function.^[5]^ This suggests that there may be changes in peripheral factors affecting oxygen transport, however, this must be further elucidated. Direct negative effects of chemotherapy on skeletal muscles are documented in animal studies, where anthracyclines impair force-generating capacity and mitochondrial function in skeletal muscles.^[6–9]^ These findings are supported by analysis on muscle biopsies from patients with prostate cancer,^[10]^ testicular cancer,^[11]^ and in 2 small-scale studies on patients with breast cancer undergoing chemotherapy.^[12,13]^ The effects of treatment with taxanes on skeletal muscle are less studied. However, muscle and joint pain, referred to as “taxane acute pain syndrome”^[14]^ is commonly reported and occurs about 24 to 48 hours after treatment and lasts for about 5 to 7 days.^[15]^ Altogether, previous studies confirm the deleterious effects of chemotherapy on muscle size, mitochondrial structures, and muscle function. An alternative reason for these muscle changes could be reduced levels of physical activity, which is common in patients with cancer.^[16]^ Epidemiological studies have found that patients receiving chemotherapy decrease their level of physical activity during treatment, and this reduction may still be present after the end of the treatment period.^[17,18]^ Regardless of cause, loss of skeletal muscle mass has been associated with reduced physical functioning and increased toxicity, that is poor tolerance to chemotherapy, and consequently worse prognosis.^[19,20]^

Over the past decades, physical activity has emerged as an important factor to improve cancer outcomes and international guidelines recommend patients to engage in regular physical activity.^[21]^ In women with breast cancer, physical exercise (i.e., planned, structured physical activity aiming to improve/sustain physical function and fitness^[22]^), has been shown to reduce the loss of muscle strength, which is commonly observed during and/or after treatment.^[23]^ Possible protective effects of physical exercise on skeletal muscle during chemotherapy for breast cancer have only been studied in 1 previous study. Mijwel and colleagues^[13]^ showed that participating in a training program that combined high-intensity-intervals with either resistance training (RT-HIIT) or moderate-intensity aerobic exercise (AT-HIIT) during treatment prevented a decrease in muscle fiber size in both intervention groups in comparison to the control group (patients receiving usual care).^[13]^ In addition, participants in the RT-HIIT group showed a greater increase in muscle fiber size and satellite cell (SC) content in comparison to participants in the AT-HIIT. These results indicate that RT on its own could have a potentially protective effect on skeletal muscle function and size in breast cancer patients undergoing chemotherapy. Resistance training during chemotherapy may also improve treatment efficiency, quality of life and reduce cancer-related fatigue, and other side effects of treatment,^[24,25]^ some of these improvements may be related to increased production of anti-tumor myokines in the exercising muscles.^[26]^ The effects of RT alone on muscle cellular outcomes during chemotherapy however, have not yet been investigated in women with breast cancer.

The *aim* of this study is to investigate the effects of heavy-load RT on muscle cellular outcomes in women with breast cancer undergoing (neo-)adjuvant chemotherapy.

Our hypotheses are:

1. (Neo-)adjuvant chemotherapy will reduce muscle fiber size, muscle strength, cardiorespiratory fitness, impair mitochondrial function, and increase indicators of cellular stress.
2. Resistance training during (neo-)adjuvant chemotherapy will reduce treatment side effects on muscle tissue by counteracting the negative effects on fiber size, mitochondrial function, cell stress, muscle strength and cardiorespiratory fitness.
3. Resistance training during (neo-)adjuvant chemotherapy will increase muscle and blood levels of potential antitumor myokines.

## 2 Methods

### 2.1 Study design

**Figure 1:**
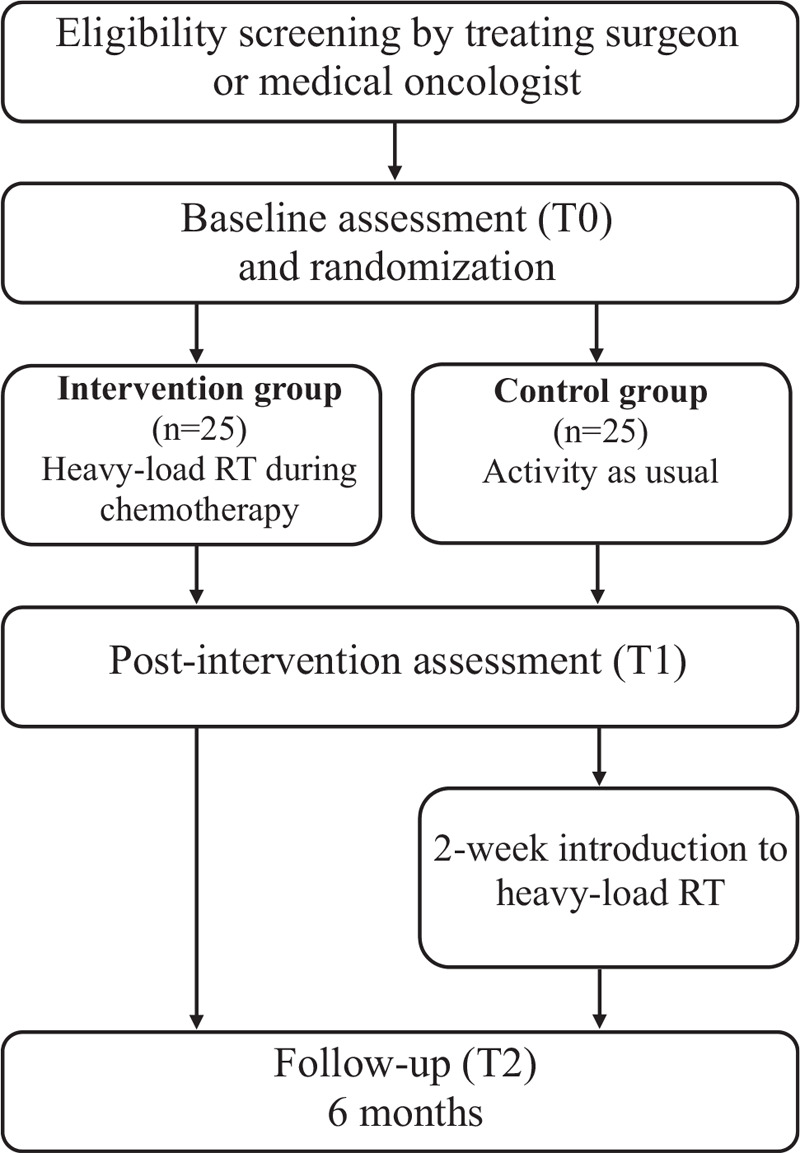
Study flowchart.

This study is a two-armed randomized controlled trial with follow-up at 6 months (Fig. 1). Data will be collected before the first cycle of chemotherapy (T0), after chemotherapy (T1), and 6 months later (T2). The primary outcome for this study is muscle fiber size. Secondary outcomes are: regulators of muscle fiber size and function, indicators of cellular stress and mitochondrial function, myokines with potential antitumor effects, muscle strength, and cardiorespiratory fitness. All outcomes are listed in Table 1.

**Table 1:** Outcomes and assessments.

	Specific variable	Assessment	T0	T1	T2
Primary outcome					
Muscle fiber size	Muscle fiber CSA	Cross-sections of muscle fiber	x	x	x
Secondary outcomes					
*Regulators of muscle fiber size and function*					
Number of myonuclei and satellite cells per muscle fiber	Myonuclei/fiber, SC/fiber	Cross-sections of muscle fiber	x	x	x
Proteins involved in muscle protein synthesis and degradation	PI3K/Akt/mTOR-pathway; including but not limited to: mTOR, P70s6k, 4EBP1, eIF4A, FOXO Ubiquitin ligase E2, LC3 (I and II), p62, myostatin, as well as ubiquitinated proteins	Western blot	x	x	x
Indicators of cellular stress	Hsp27, αB-crystalline, Hsp60, Hsp70	Western blot	x	x	x
Mitochondrial function	Mitochondrial structure	Cross-sections and whole fiber preparations of muscle biopsies	x	x	x
	CS, Cox 4 and HAD	Western blot	x	x	x
*Myokines*					
Potential anti-tumor myokines	Including, but not limited to: IL-6, IL-15, SPARC, TWEAK, IL-8, IL-10, IL-1β, IFN-γ, TNF-α, TNFR1	mRNA levels by real-time PCR analyses and protein levels by Western blot and ELISA	x	x	x
*Muscle Strength*					
	Upper extremities	1RM, chest press.	x	x	x
	Lower extremities	1RM, single leg press	x	x	x
*Cardio-respiratory fitness*					
	VO_2max_	Maximal oxygen uptake test	x	x	x
*Body composition*					
	Fat free mass, Fat mass	BodPod, BIA	x	x	x
	Body mass index	Weight and height	x	x	x
*Biomarkers of oxygen transportation, stress, inflammation, metabolic outcomes and muscle damage*					
	Hemoglobin, serum-cortisol, HbA1c, blood lipids, CRP and CK-MB	Standardized clinical measures	x	x	x
*Sociodemographic*					
	Age, partnership, number and age of children living at home, education, income, work and sick leave	Study-specific questionnaire	x		
*Lifestyle, quality of life and fatigue*					
	Dietary habits, alcohol consumption, tobacco use, motivation, self-efficacy and barriers to training. Health-related quality of life, Cancer related fatigue	Study-specific questionnaire, EORTC QLQ-C30, QLQ-BR25, MFI	x	x	x
*Physical activity*					
	Physical activity level	Sensewear Armband	x	x	x
*Disease and treatment*					
Disease specific information	Diagnosis Type, dose and side-effects of, and adherence to oncological treatment	Medical records			x
*Adverse events*					
	Adverse events occurring during exercise training sessions	Reported by coaches		x	x
	Adverse events occurring during muscle biopsy sampling	Reported by technicians	x	x	x

4EBP1 = Eukaryotic translation initiation factor 4E-binding protein 1, BIA = Bioelectrical impedance analysis, CK-MB = creatine kinase-myocardial band, Cox 4 = cytochrome c oxidase subunit 4, CRP = C-reactive protein, CS = citrate synthase, CSA = cross-sectional area, eIF4A = eukaryotic initiation factor-4A, EORTC QLQ-C30 = European Organization for the Research and Treatment of Cancer Quality of Life Questionnaire Core 30, HAD = 3-hydroxyacyl-CoA-dehydrogenase, Hsp27, 60 and 70 = heat shock protein 27, 60 and 70, IFN-γ = Interferon gamma, IL-6, -8, -10, -15, -1β = Interleukin- 6, -8, -10, -15 and 1beta, MFI = Multidimensional fatigue inventory, mTOR = mechanistic target of rapamycin, p62 = ubiquitin-binding protein p62, P70s6k = ribosomal protein S6 kinase, QLQ-BR25 = The European organization for research and treatment of cancer quality of life questionnaire, SC = satellite cell, SPARC = secreted protein acidic and rich in cysteine (Osteonectin), T0 = before start of chemotherapy and training intervention, T1 = after chemotherapy and training intervention, T2 = 6-month follow-up, TNF-α = tumor necrosis factor alpha, TNFR1 = tumor necrosis factor receptor 1, TWEAK = TNF-related weak inducer of apoptosis, VO^2^max = maximal oxygen uptake.

### 2.2 Participant recruitment and eligibility criteria

Women recently diagnosed with breast cancer will be recruited from Uppsala University Hospital.

Eligibility criteria are:

1. women diagnosed with stage I-III breast cancer,
2. >18 years old,
3. literate in Swedish,
4. scheduled to undergo (neo-)adjuvant chemotherapy with a combination of taxanes and anthracyclines or only 1 of these treatments.

Exclusion criteria are:

1. unable to perform basic activities of daily living,
2. cognitive disorders or severe emotional instability,
3. other disabling comorbidities that might hamper physical training (e.g., severe heart failure, chronic obstructive pulmonary disease, orthopedic conditions, and neurological disorders).

Eligibility will be assessed by treating surgeons or medical oncologists, and all eligible women will receive written information. Women who meet the inclusion criteria will be provided with further information about the study and written informed consent will be obtained by research staff. Based on the experience gained from on-going studies and previous relevant studies within the research group, an inclusion rate of 35% to 40% can be expected. The study was initiated in December 2018 and with the expected inclusion rate; the last participants will complete the intervention during the second half of 2021.

### 2.3 Sample size

The power calculation is based on results from strength training effects in prostate cancer patients on androgen deprivation therapy, the Physical Exercise and Prostate Cancer trial (PEPC trial).^[10]^ With a similar effect on muscle fiber size (+898 ± 840 μm^2^), we need 10 participants in each group to get a statistical power of 80% in the present study, to further enhance the power up to 95%, we need 16 participants in each group. Consequently, 50 participants (25 in each arm) will be recruited to account for drop-outs during the intervention. The power calculation is based on the PEPC trial^[10]^ but findings from the study by Mijwel and colleagues^[13]^ strongly support similar expectations in breast cancer patients on chemotherapy.

### 2.4 Randomization

After completion of baseline measurements, participants will be randomized by research staff in a 1:1 ratio to either the *intervention group* (supervised heavy-load RT during chemotherapy treatment) or to the *control group* (activity as usual during chemotherapy treatment), stratified on treatment (neo-adjuvant or adjuvant treatment). Sealed envelopes will be used to conceal the group allocation.

### 2.5 Intervention

Participants in the intervention group will perform supervised heavy-load RT twice a week from the week following T0 and throughout the course of treatment with chemotherapy, approximately 16 weeks. Sessions will be performed at a public gym and led by trained coaches. The following 6 exercises will be included in the program: seated leg-press, seated chest-press, seated leg-curl, seated row and seated leg-extension performed in machines and seated overhead-press using dumbbells. Completed training (weight lifted, number of repetitions and sets) and rating of perceived exhaustion will be documented by the participants in training logbooks. The scheduled timeframe of the training is described in Table 2. Briefly, the first 2 weeks of the program represent familiarization to the training protocol, tests, and Omni-scale for self-reported perceived exertion.^[27]^ During this period, the participants will perform exercises at a light load. After the first 1 RM-test (1 repetition maximum test), training will progress in sets and training load before testing of 6- and 10 RM which will provide the participants with individualized loads. Rest periods between sets will be 2 (6 RM training load) and 1 minute (10 RM training load) for the 2 different sessions, respectively. The training load will be adjusted throughout the intervention period. Participants in the control group are encouraged to continue with their activity as usual that is, maintain their habitual physical activity level and not initiate RT during chemotherapy. To increase interest in participation, controls will be invited to a 2-week introduction to the same resistance-training program as the intervention group following completion of chemotherapy and offered a 12-month membership at a local gym, free of charge.

**Table 2:** Overview of the resistance training intervention.

Week	Training session 1	Training session 2
1	1×20 sub-maximal load Focus on correct technique	1×20 sub-maximal load Focus on correct technique
2	2×10 sub-maximal load	1RM-test
3–4	2×10 sub-maximal load	2×10 sub-maximal load
5	Test of 10RM	Test of 6RM
6	2×10 RM	2×6 RM
7–15	3×10 RM	3×6RM
16	3×10RM	1RM-test

RM = repetition maximum.

### 2.6 Physical training logbook

To control for physical activity level during the intervention all participants will be instructed to keep a logbook of all physical training. Participants in the intervention group will be instructed to record all endurance training lasting more than 10 minutes with a perceived exertion of 15 (strenuous) or higher on the Borg-scale^[28]^ and any additional RT session performed. Participants in the control group are instructed to record all endurance training lasting more than 10 minutes with a perceived exertion of 15 (strenuous) or higher on the Borg-scale (29) and all resistance-training sessions.

### 2.7 Muscle biopsy procedure

Muscle biopsies are obtained from the mid-section of *m. vastus lateralis* under local anaesthesia (Xylocain adrenaline, 10 mg ml^−1^ + 5 μg ml^−1^, AstraZeneca, Södertälje, Sweden). Briefly, a 1 to 2 cm incision will be made in the skin and the fascia of *m. vastus lateralis*. Biopsies are collected using a 6 mm Pelomi-needle (Albertslund, Denmark; Bergström technique) with manual suction to obtain muscle samples (≈200 mg). Biopsies will be rinsed in ice-cold saline 0.9% NaCl (Braun, Melsungen, Germany) and carefully dissected free of visual fat, connective tissue and blood. All pieces but 1 will be frozen in isopentane, precooled on dry ice, and stored at −80°C for later analysis. The last piece (≈10 mg) will be transferred to 500 μl RNA*later* Stabilization Solution (Invitrogen, Carlsbad, CA) and stored at +4°C for at least 24 hours before being transferred to −20°C for long-time storage.

### 2.8 Primary outcome

#### 2.8.1 Muscle fiber size

Muscle fiber size, measured as muscle fiber cross-sectional area (CSA) represents the primary muscle cellular outcome. Muscle fiber CSA will be measured using immunohistochemistry in cross-sections of muscle biopsies. Briefly, transverse serial sections of the muscle biopsy (8 μm thick) will be cut using a cryostat microtome at −22°C and mounted on glass slides. Serial cross-sections will be immunohistochemically stained for fiber types (type I, type IIa, and IIx) for CSA measure. Muscle fiber CSA will be measured for the different phenotypes separately. Assessors will be blinded to each participant's allocation.

### 2.9 Secondary outcomes

#### 2.9.1 Regulators of muscle fiber size

Secondary muscle cellular outcomes reflecting regulators of muscle fiber size are

1. number of myonuclei per muscle fiber,
2. number of SCs per muscle fiber,
3. proteins involved in muscle protein degradation (muscle breakdown), and
4. regulators of muscle protein synthesis (local growth factors).

Muscle fiber myonuclear and SC content per muscle fiber will be measured using immunohistochemistry in cross-sections of muscle biopsies as previously described. Myonuclei- and SCs content per muscle fiber will be assessed for the different phenotypes separately (type I, type IIa and IIx). Regulators of muscle fiber size, that is proteins involved in muscle protein synthesis and protein degradation will be measured using Western blot analysis in muscle homogenate. See Table 1 for details.

#### 2.9.2 Regulators of muscle fiber function, cellular stress, and mitochondrial function

Proteins involved in protection against cellular stress (Heat Shock proteins: Hsp 27, αB-crystalline, Hsp 60, and Hsp 70), as well as enzymes involved in mitochondrial function (CS, Cox 4, and HAD) will be assessed in muscle homogenate using Western blot analysis. In addition, mitochondrial structure will be studied in cross-sections and whole fiber preparations of muscle biopsies using immunohistochemistry.

#### 2.9.3 Myokines with potential antitumor effects

Exploratory analyses on the effects of the RT on the expression levels of myokines, previously proposed to have an antitumor effect, will be conducted. Relevant targets, including, but not limited to, IL-6, IL-15, SPARC, and TWEAK will be evaluated on the mRNA level by real-time PCR analyses (RNA extracted from biopsies) and protein level by Western blot and enzyme-linked immunosorbent assays (ELISA) (muscle and blood samples). Blood samples will be obtained by venipuncture and participants are asked to avoid smoking and alcohol and not to engage in any strenuous physical activity 24 hours before the blood sample. IL-6, IL-8, IL-10, IL-1β, IFN-γ, TNF-α, TNF1R will be measured using ELISA based methods. Frozen sera will be saved for further analyses that can be included later.

#### 2.9.4 Muscle strength 

Maximal upper- and lower- extremity muscle strength will be measured as 1 RM in seated chest-press and seated single-leg press. To secure the validity of the 1 RM tests, all participants will undertake a 2-week familiarization period prior to these assessments (both participants in the intervention group as well as participants allocated to the control group).

#### 2.9.5 Cardiorespiratory fitness

Cardiorespiratory fitness will be measured as maximal oxygen uptake during maximal walking/running until exhaustion on a treadmill using a modified Balke-protocol^[29]^ starting at 4 km/hour with an incline of 2%. The inclination increases with 1% every minute until reaching 12%, from which the speed increases 0.5 km/hour per minute until exhaustion. Oxygen consumption and minute ventilation will be measured continuously using an oxygen analyzer (Vmax Encore system, Carefusion, CA). Heart rate will be measured using a heart rate monitor (Polar RS400, Kempele, Finland) and self-perceived exertion will be recorded using a standardized Borg-scale.^[28]^

### 2.10 Additional outcome measures

#### 2.10.1 Body composition

Fat-free mass and fat mass will be measured with air displacement plethysmography (Bod Pod, Life Measurement System Inc., Concord, CA), and bioelectrical impedance analysis (BIA, TANITA BC-420MA, Tanita Corporation, Japan). Bodyweight is measured using BIA and body height is measured with a stadiometer, body mass index (BMI: weight/(height^2^)) is then calculated.

#### 2.10.2 Biomarkers of oxygen transportation, stress, inflammation, metabolic outcomes, and muscle damage

Blood samples will be obtained as described earlier and analyzed for levels of hemoglobin, serum-cortisol, long-term average blood glucose (hemoglobin A1c [HbA1c]), blood lipids, C-reactive protein (CRP), and creatine kinase-myocardial band (CK-MB) using standardized clinical measures.

#### 2.10.3 Sociodemographic, lifestyle, quality of life, and fatigue

Age, partnership, number and age of children living at home, education, income, work and sick leave will be assessed using a study-specific questionnaire. Dietary habits, alcohol consumption, tobacco use, motivation, self-efficacy, and barriers to exercise are self-reported using a study-specific questionnaire. The EORTC QLQ-C30^[30]^ and diagnosis-specific modules (QLQ-BR25 for breast cancer) will be used to assess health-related quality of life. Cancer-related fatigue will be assessed using the MFI.^[31]^

#### 2.10.4 Physical activity level

Physical activity level will be assessed by SenseWear Armband (SWA) Mini (BodyMedia Inc., Pittsburgh, PA). All participants will be instructed to wear the SWA for 7 consecutive days. Only valid days with at least 80% wearing time will be included in the analyses. SWA data will be downloaded and analyzed with software developed by the manufacturer (SenseWear Professional Research Software Version 8.1, BodyMedia Inc., Pittsburgh, PA). During this week, participants will self-report their physical activity level (time spent, types of activity, and its intensity), time spent sleeping, time spent in sedentary behaviors (sitting, lying down during waking hours), use of medications for nausea or pain, and potential sick leave or reduced work capacity in logbooks.

#### 2.10.5 Disease and treatment

Information about type and dose of oncological treatment, stage of disease, co-morbidity, central catheter (including type, insertion/removal date, and complications) and treatment side effects will be collected from medical records. Chemotherapy treatment completion rates will be calculated as described by Longo et al.^[32]^

#### 2.10.6 Adverse events

Adverse events caused by the exercise are registered by the coaches. Grade 1 (e.g., muscle strain) means that the participant has to terminate the ongoing specific exercise but can continue with the exercise session. Grade 2 (e.g., fall in blood pressure) means that the participant must terminate the exercise session. Any severe adverse events (e.g., fracture) are reported directly to the PI and managed by healthcare. Adverse events from muscle biopsy procedure will also be registered.

### 2.11 Statistical analyses

Data will be analyzed according to the intention-to-treat principle. Analyses will include standard descriptive statistics, *t* tests, correlation, regression and two-way repeated-measures ANOVA or the comparable nonparametric test as necessary to examine differences between and within groups, at T0, T1, and T2. In addition, a per-protocol analysis, that is, adherence to the protocol, will be conducted. Should imbalances in important variables be detected, sensitivity analyses will also be added including these as covariates in the model. All data will be cleaned and quality-checked before analysis.

### 2.12 Ethics and dissemination

The present study has been approved by the Regional Ethical Review Board in Uppsala, Sweden (Dnr 2016/230/2) and registered in ClinicalTrials.gov (NCT04586517), registered on 14 October, 2020. The study will be performed in accordance with the Declaration of Helsinki and written informed consent according to GDPR guidelines will be obtained from the participants. Collections of biological material as blood and tissue samples will be stored according to the Regional Biobank in Uppsala. Results will be disseminated through presentations at scientific meetings, publications in peer-reviewed journals, social media, and patient organizations. A data sharing plan has been established, where de-identified individual data on participant characteristics and main study outcomes will be made available on reasonable request once the results are published.

## 3 Discussion

The present study aims to investigate the effects of a heavy-load resistance-training program on skeletal muscle cellular outcomes in women diagnosed with breast cancer undergoing chemotherapy. The results are expected to provide insights about the mechanism involved in side effects of chemotherapy on skeletal muscle. Such knowledge can be used to design effective physical exercise programs to support individuals with breast cancer during and following chemotherapy and possibly reduce long-lasting side effects such as impairments in physical function, fatigue, and risk for comorbidities.

To our knowledge, this will be the first randomized controlled study evaluating heavy-load RT during (neo-)adjuvant chemotherapy treatment in breast cancer patients to explore its effects on muscle cellular outcomes in a relatively larger study population in comparison to previous studies.^[13,33,34]^ Although the primary outcome of this present study is muscle fiber CSA, we are also including a wide range of biological measurements, including specific proteins involved in skeletal muscle hypertrophy/degradation and regulators of muscle fiber function. These analyses will provide further insight into the underlying mechanism through which chemotherapy affects muscle tissue and how RT could be used as a therapeutic measure to counteract immediate and long-term treatment side effects. As exercise has been suggested to have an antitumor effect^[26]^ regular exercise could have a positive effect on the recurrence rate. In this study, we will investigate changes in myokines with potential antitumor effect in both muscle tissue and blood and these results will give valuable insight to exercise-induced antitumor mechanisms. However, the possible effect on cancer recurrence rate has to be further explored in future longitudinal studies as our study will only include follow-up measures at 6 months post end of treatment. Importantly, the feasibility of the training program has been confirmed by a previous study (Phys-Can study)^[29]^) in which ∼75% of participants completed the 6-month training program with an exercise adherence of approximately 67%.

In summary, previous research underlines the potential of physical exercise during cancer treatment on improving outcomes such as physical function, mental health, fatigue, and quality of life in women with breast cancer.^[35–37]^ However, research on the specific cellular effects of RT during chemotherapy is scarce. This study will provide important knowledge regarding the effects of heavy-load RT on skeletal muscle cellular outcomes. In addition, increased knowledge regarding the effects of RT on a selection of antitumor myokines as well as muscle strength and cardiorespiratory fitness may be provided.

## Acknowledgments

We would like to thank the staff at the surgical department, the oncology department and the Rudbeck laboratory, Uppsala University Hospital, for invaluable help with recruitment of participants and for providing facilities. We would also like to thank Friskis & Svettis Väderkvarn, Uppsala, for providing training facilities.

## Author contributions

**Conceptualization:** Karianne Vassbakk-Svindland, Anna Henriksson, Birgitta Johansson, David Kudrén, Henrik Lindman, Fredrik Wärnberg, Sveinung Berntsen, Ingrid Demmelmaier, Karin Nordin, Truls Raastad.

**Data curation:** Emelie Strandberg, Karianne Vassbakk-Svindland, Olav Vikmoen, Tim Schauer.

**Funding acquisition:** Karin Nordin, Truls Raastad.

**Investigation:** Emelie Strandberg, Karianne Vassbakk-Svindland, Olav Vikmoen, Tim Schauer.

**Methodology:** Anna Henriksson, Birgitta Johansson, David Kudrén, Henrik Lindman, Fredrik Wärnberg, Sveinung Berntsen, Ingrid Demmelmaier, Karin Nordin, Truls Raastad.

**Project administration:** Emelie Strandberg, Karianne Vassbakk-Svindland.

**Supervision:** Ingrid Demmelmaier, Karin Nordin, Truls Raastad.

**Writing – original draft:** Emelie Strandberg, Karianne Vassbakk-Svindland.

**Writing – review & editing:** Emelie Strandberg, Karianne Vassbakk-Svindland, Anna Henriksson, Birgitta Johansson, Olav Vikmoen, David Kudrén, Tim Schauer, Henrik Lindman, Fredrik Wärnberg, Sveinung Berntsen, Ingrid Demmelmaier, Karin Nordin, Truls Raastad.
